# Dynamics Contributions to the Growth Mechanism of Ga_2_O_3_ Thin Film and NWs Enabled by Ag Catalyst

**DOI:** 10.3390/nano9091272

**Published:** 2019-09-06

**Authors:** Badriyah Alhalaili, Ryan Bunk, Ruxandra Vidu, M. Saif Islam

**Affiliations:** 1 Nanotechnology and Advanced Materials Program, Kuwait Institute for Scientific Research, Safat 13109, Kuwait; 2 Electrical and Computer Engineering, University of California, Davis, CA 95616, USA; rjbunk@ucdavis.edu (R.B.); ruxiev@gmail.com (R.V.); sislam@ucdavis.edu (M.S.I.); 3 The Faculty of Materials Science and Engineering, University of Politehnica of Bucharest, 060042 Bucharest, Romania

**Keywords:** thin film, nanowires, Ga_2_O_3_, quartz, thermal oxidation, silver catalyst

## Abstract

In the last few years, interest in the use of gallium oxide (Ga_2_O_3_) as a semiconductor for high power/high temperature devices and UV nano-sensors has grown. Ga_2_O_3_ has an enormous band gap of 4.8 eV, which makes it well suited for applications in harsh environments. In this work, we explored the effect of Ag thin film as a catalyst to grow gallium oxide. The growth of gallium oxide thin film and nanowires can be achieved by heating and oxidizing pure gallium at high temperatures (~1000 °C) in the presence of trace amounts of oxygen. We present the results of structural, morphological, and elemental characterization of the β-Ga_2_O_3_ thin film and nanowires. In addition, we explore and compare the sensing properties of the β-Ga_2_O_3_ thin film and nanowires for UV detection. The proposed process can be optimized to a high scale production Ga_2_O_3_ nanocrystalline thin film and nanowires. By using Ag thin film as a catalyst, we can control the growth parameters to obtain either nanocrystalline thin film or nanowires.

## 1. Introduction

In recent years, gallium oxide (Ga_2_O_3_) has become one of the most significant materials that can operate in harsh conditions such as automobiles engines, flame monitoring, space communications, and detection of missiles. This material has a band-gap of 4.8 eV, a high melting point of 1900 °C, and excellent electrical and photoluminescence properties [1,2,3]. Ga_2_O_3_ has the potential to replace Si, SiC, GaN/AlGaN in high power applications due to its superior breakdown voltage and low on-resistance [4]. Additionally, the fundamental band gap is above the upper range of the terrestrial solar spectrum; hence, Ga_2_O_3_ is expected to be intrinsically solar-blind as a photodetector [5,6]. Therefore, it is considered the best choice for visible-blind (UV) photodetectors and power electronics in harsh environments.

Although Ga_2_O_3_ is a desired material in the semiconductor industry, the cost of Ga_2_O_3_ wafers is still prohibitively high and methods for the top-down and bottom-up fabrication of Ga_2_O_3_ nanostructures are still immature. Hence, a wide variety of methods to grow Ga_2_O_3_ thin film and nanowires have been explored, such as thermal oxidation [7,8], vapor–liquid–solid growth [9], pulsed laser deposition [10], sputtering [11], thermal evaporation [12,13,14], molecular beam epitaxy [15], laser ablation [16], arc-discharge [17], carbothermal reduction [18], microwave plasma [19], metalorganic chemical vapor deposition [20], and the hydrothermal method [21,22]. In this work, the thermal oxidation process was performed to explore the effect of silver thin film as a catalyst to enhance the growth of Ga_2_O_3_. This process is one of the less expensive techniques that operate at a high temperature. In addition, Ag helps to increase the growth of Ga_2_O_3_ due to the self-assembly growth of nanocrystalline grains [23]. Consequently, the presence of an effective catalyst that has the ability to tolerate higher temperatures than its melting point could be a valuable factor in spontaneously increasing the reaction rate of the growth mechanism.

Silver nanoparticles (Ag NPs) have been explored as a possible catalyst for Ga_2_O_3_ nanowire growth [9,24,25]. In addition, silver has been used as a photocatalyst and doping material for different materials such as MoS_2_, SnO_2_ nanostructures, and TiO_2_/SnO_2_ nanocomposite [26,27,28]. To date, the effect of Ag thin film as a catalyst on Ga_2_O_3_ growth of thin film and nanowire at different growth parameters have not been explored. Because of oxygen’s high diffusivity and solubility at higher temperatures, more atmospheric O_2_ accumulation leads to effective transport into the Ga and enhance spontaneous Ga_2_O_3_ growth. However, other metals used as catalysts either lack solubility (Au) or diffusivity (Fe).

We propose a simple and inexpensive thermal oxidation process to produce Ga_2_O_3_ thin film and nanowires using 5 nm and 300 nm Ag thin film as a catalyst patterned on a quartz substrate. Because the influence of the oxidation parameters (i.e., annealing temperature, time, and oxygen concentration) and Ag film thickness on Ga_2_O_3_ growth had not been comprehensively investigated, various parameters of Ga_2_O_3_ growth due to silver presence were examined in this work. Different characterization techniques, such as X-ray diffraction (XRD), scanning electron microscopy (SEM), energy dispersive X-ray spectroscopy (EDS), and electrical measurements have been performed in this study to explore the growth mechanism of thin film and nanowire of Ga_2_O_3_.

## 2. Materials and Methods

Quartz substrates used in this experiment were 500 µm thick and 15 mm in diameter. First, quartz substrates were cleaned with acetone and ethyl alcohol, rinsed with deionized water, and then dried with an N_2_ blow gun to remove the excess residues. To investigate the effect of Ag NPs during the growth of Ga_2_O_3_, two different thicknesses of Ag thin film such as 5 nm and 300 nm were deposited on the samples with a Lesker sputtering system by using a shadow mask. This patterned surface offers the opportunity to investigate concomitantly the growth of gallium oxide on Ag-coated and uncoated surfaces. We have also compared the results with uncoated samples grown under the same conditions.

**Figure 1:**
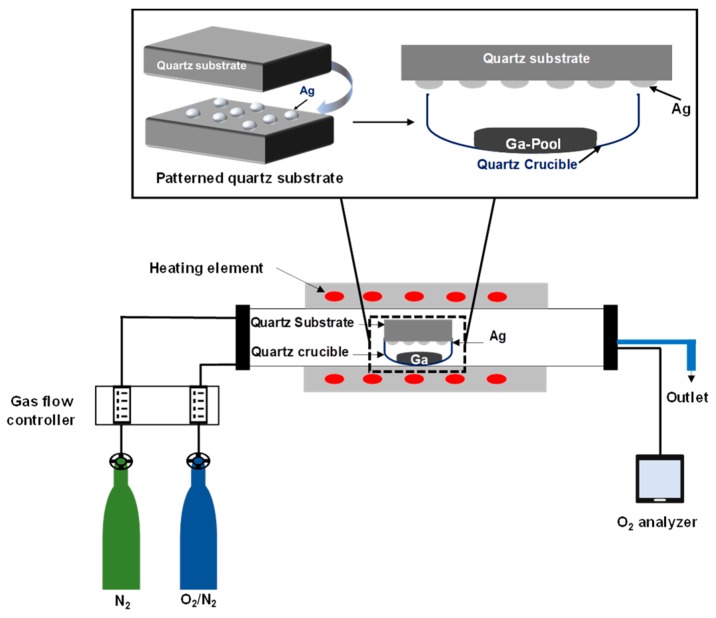
Schematic of the sample set-up inside the furnace, which was used to study the growth mechanism of gallium oxide on a quartz substrate coated with a 5 nm and 300 nm patterned Ag film sputtered by a shadow mask. The quartz substrate was placed over the Ga source, having the patterned surface facing downward over the Ga source. The Ga liquid pool is in a quartz crucible at a distance of ~10 mm from the sample.

Then, the sample was loaded into a quartz crucible which was placed into an OTF-1200X-50-SL horizontal alumina tube furnace made by MTI Corporation (MTI Corporation, Richmond, CA, USA). A controlled stream of O_2_ and N_2_ mixture gases flowing through the quartz tube was performed to trace the concentration of oxygen molecules by using an oxygen analyzer (EQ-W1000-LD, MTI Corporation, Richmond, CA, USA) made by MTI Corporation. The samples were heated up to 1000 °C for 60 min, with a controlled heating and cooling rate of 17 °C per second. The temperature was regulated during heating, cooling, and holding by use of a proportional-integral-derivative (PID) controller. The entire process occurred in a flow of 20 sccm nitrogen atmosphere. The concentration of O_2_ gas was varied by adjusting the gas flow controller, which controlled the ratio of O_2_ to N_2_ gas. The background oxygen concentration was determined to be from 88 ppm to 280 ppm. Samples were positioned so that the silver patterned surface faced the gallium pool. The distance between the substrate and the gallium pool was 10 mm. Then, the quartz crucible was inserted inside the furnace for the oxidation process. Figure 1 illustrates the set-up of the sample inside the furnace, which was used to grow gallium oxide on an Ag catalyst patterned surface.

## 3. Results & Discussion

### 3.1. X-ray Diffraction

**Figure 2:**
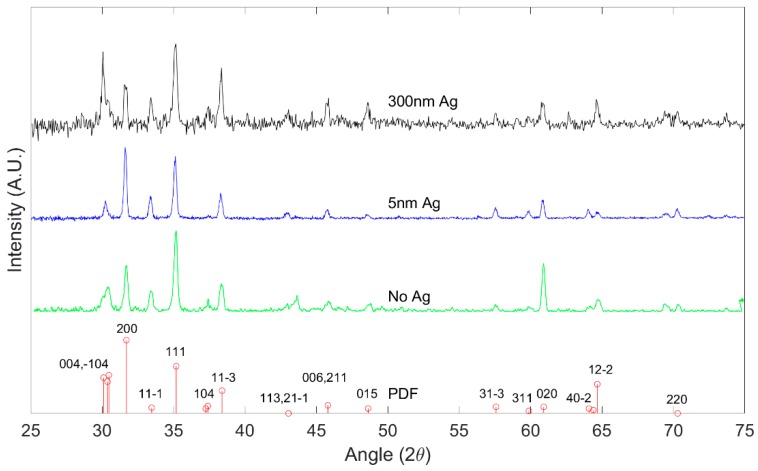
X-ray diffraction (XRD) pattern of β-Ga_2_O_3_ for different Ag thickness at 1000 °C, indexed in comparison to PDF 00-041-1103.

X-ray Diffraction was performed with a Panalytical XPert PRO Diffractometer (Malvern Panalytical, Netherlands). Figure 2 shows the peak positions of the X-ray diffraction pattern of grown Ga_2_O_3_ on quartz substrates oxidized at 1000 °C from the main planes in the crystalline β-Ga_2_O_3_. The results are consistent with polycrystalline β-Ga_2_O_3_ as referenced against International Center for Diffraction Data (ICDD) standard PDF 00-041-1103 [29]. All major peaks of the β-Ga_2_O_3_ phase are seen to be present in the diffraction data, which strongly indicates the presence of β-Ga_2_O_3_.

### 3.2. SEM Characterization

**Figure 3:**
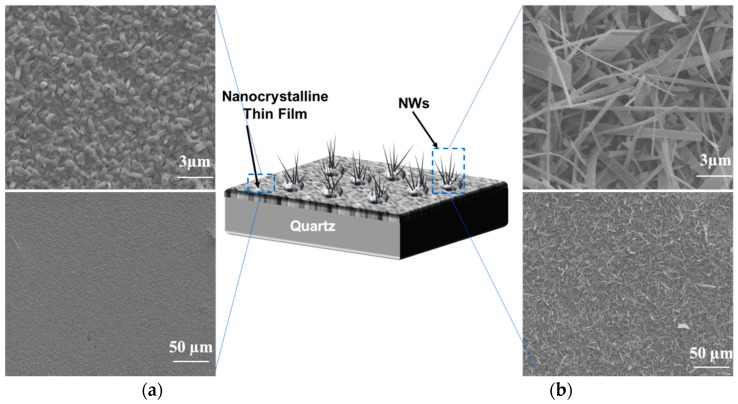
Scanning electron microscopy (SEM) images of 5 nm Ag-coated and uncoated areas acquired in the regions indicated in the illustration (middle). (**a**) Thin film. (**b**) nanowires.

**Figure 4:**
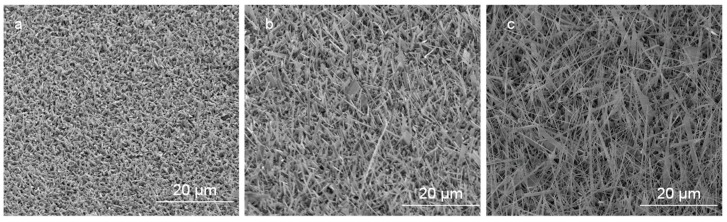
SEM images of uncoated and Ag-coated areas acquired in the regions indicated in the illustration. (**a**) nanocrystalline thin film of Ga_2_O_3_ obtained by the indirect effect of the sample patterned by Ag catalyst within the uncoated area. (**b**) Ga_2_O_3_ NWs under the presence of 5 nm Ag. (**c**) Ga_2_O_3_ NWs under the presence of 300 nm Ag.

The morphology of Ga_2_O_3_ nanowires and thin films that were concomitantly grown on Ag-patterned quartz at 1000 °C is shown in Figure 3. The Ag-patterned quartz surface shows distinct nanowires and thin film morphologies of Ga_2_O_3_ that were grown concurrently on Ag-coated and uncoated areas, as illustrated in Figure 3. Ag-patterned areas have been converted into dense nanowires while the uncoated areas became a nonuniform coating of Ga_2_O_3_ thin film. In the Ag-patterned areas, the nanowire density was increased, and their diameter was reduced depending on the Ag thin film thickness. The diameter of Ag-free growth was about 150–270 nm. When 5 nm and 300 nm Ag thin film was used, the NWs diameters were 120–160 and 80–130 nm, respectively. Even though the sample coated with 300 nm Ag has reduced the diameter of the nanowire and increase its density (Figure 4), we consider that presenting the sample coated with 300 nm Ag does not bring anything new. Hence, the results obtained for 5 nm Ag are further discussed and compared to the 300 nm Ag.

**Figure 5:**
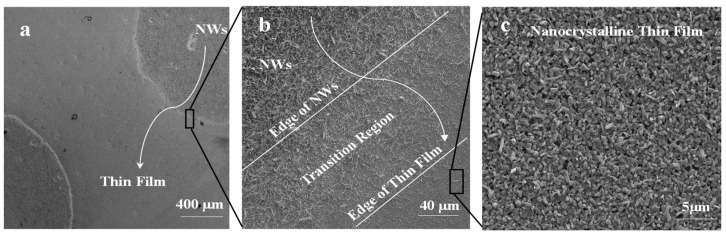
Ga_2_O_3_ NWs grown on quartz at 1000 °C. (**a**) Top view of Ag circle pattern which was converted into dense NWs and surrounded by a nonuniform coating of thin film. (**b**) Interface of Ag-coated area and uncoated region shows a gradual transition of NW growth to thin film. (**c**) Nanocrystalline thin film of Ga_2_O_3_ is due to surface diffusion of silver nanoparticles (Ag NPs).

Figure 5 shows SEM images of the 5 nm Ag patterned quartz circles, which were converted into dense and long Ga_2_O_3_ nanowires; however, the bare quartz was converted into Ga_2_O_3_ nanocrystalline thin film. The area between Ag circles and the uncoated surface shows a gradual morphology from nanowires to thin film due to the lateral diffusion of Ag atoms. The lateral diffusion and distribution of Ag enhance the growth of nanowires and increase the process rapidly and spontaneously in the area that was intentionally coated with Ag thin film. In addition, the presence of Ag affects the uncoated area and enhances nucleation of nanocrystalline thin film of Ga_2_O_3_. Due to the self-diffusion of Ag atoms, more oxygen molecules will enhance the dissolution of gallium suboxide (Ga_2_O) in the liquid Ag and deposit Gallium (III) oxide (Ga_2_O_3_) through crystal nucleation on the bare quartz by vapor–liquid–solid (VLS) growth mechanism to form a nanocrystalline thin film [9].

Different types of self-diffusion can affect the distribution of Ag atoms on the surface. Surface diffusion has the lowest activation energy (0.39 eV) compared to the activation energy of grain boundary diffusion (0.99 eV) and volume diffusion (1.98 eV) [30]. Therefore, during the surface diffusion process, Ag atoms can move easily with no restriction and spread on the surface. At a low activation energy and high diffusion rate, the flux of atoms increases as the concentration gradient and temperature increase. It has been observed that as the temperature increases, the density of Ag nanoparticles also increases [31]. However, grain boundary diffusion and surface diffusion are predictable mechanisms in the oxidation process. Surface diffusion increases as the duration of thermal annealing increases within a fixed temperature [32,33].

### 3.3. FIB/EDS Characterization

**Figure 6:**
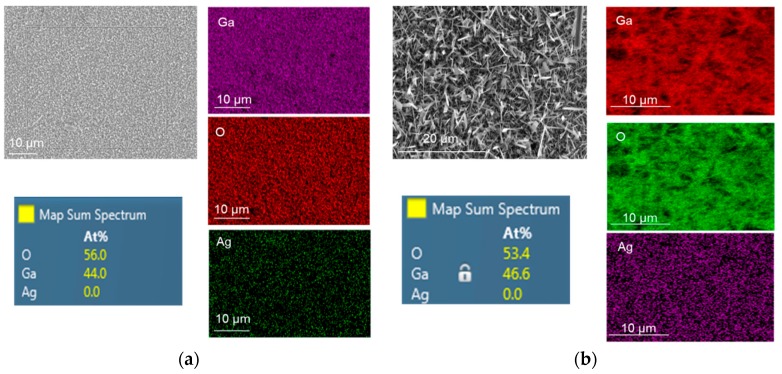
Energy dispersive spectroscopy (EDS) mapping of Ga_2_O_3_ grown at 1000 °C on quartz surface: (**a**) Nanocrystalline film of Ga_2_O_3_. (**b**) Ga_2_O_3_ nanowires catalyzed by 5 nm Ag. Longer and denser NWs were achieved when Ag was used as a catalyst.

A focused ion beam (FIB) equipped with X-MaxN 50 mm^2^ energy dispersive spectroscopy (EDS) from Oxford Instruments was used to perform elemental and chemical microanalyses on the samples. EDS mapping results of oxidized Ga without and with 5 nm Ag catalyst at 1000 °C for 60 min are presented in Figure 6. It is obvious that the atomic percentage of Ga was almost doubled under the presence of Ag catalyst. Due to the EDS detection limit, the presence of Ag atoms was not detected even though the morphology of grown Ga_2_O_3_ on Ag was greatly influenced compare to the Ag-free Ga_2_O_3_. The growth of gallium oxide is based on the vapor–liquid–solid (VLS) process [9]. Ga_2_O vapor was used as the gas source and the Ag catalyst served as the active site, leading to the growth of solid Ga_2_O_3_ at 1000 °C.

### 3.4. Scanning Transmission Electron Microscopy (STEM)

**Figure 7:**
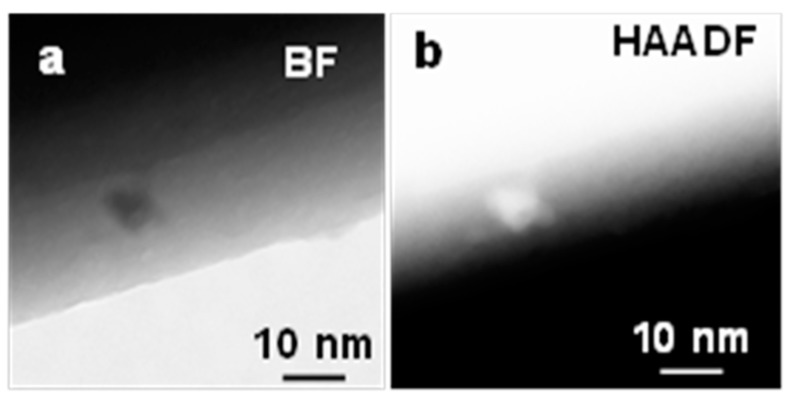
(**a**) Bright field (BF) (**b**) high-angle annular dark-field (HAADF) STEM images of Ag NPs at the interface of Ga_2_O_3_ nanowire growth on quartz substrate at 1000 °C. The NP was bright due to z-contrast.

Figure 7 shows a STEM image of the bright field (BF) and high-angle annular dark field (HAADF) of Ga_2_O_3_ nanowire grown on the quartz coated with 5 nm Ag and oxidized at 1000 °C. The β-Ga_2_O_3_ nanowire contained a few NPs with diameters between 5–10 nm that decorated the surface. The bright field showed dark NPs due to diffraction contrast since they are crystalline. Furthermore, HAADF was performed to capture z-contrast of Ag NPs. The NPs appear much brighter which suggest the average composition is heavier. Thus, the HAADF image of NPs has low noise and was expected to show a solid bright signal due to the high mass of the nanoparticles. This could suggest that these NPs could be Ag NPs.

### 3.5. Factors Effecting the Ga_2_O_3_ Growth

- Silver Thin Film Thickness

Two different thicknesses of silver thin film have been selected to explore their morphological effects onto the growth kinetics under Ag presence, i.e., 5 nm and 300 nm, as shown in Figure 3b and Figure 6b, respectively. Ag thin films could be an alternative catalyst technique to nanoparticles for Ga_2_O_3_ growth since Ag plays a critical role in increasing the nanocrystalline nucleation of the film and forming denser, longer, and thinner nanowires as the temperatures increase above the silver melting point.

**Figure 8:**
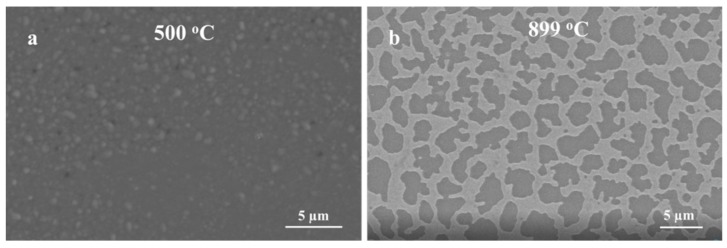
SEM images of the annealed Ag thin film of different thickness on quartz. (**a**) 5 nm Ag, (**b**) 300 nm Ag.

Obviously, the growth kinetics are highly influenced by the thickness of the Ag thin film and high oxidation temperatures. When Ag thin film was 5 nm, silver film dewets and forms nanoparticle-like structures [34]. It has been shown that 5 nm and 10 nm Ag thin film dewets very fast at a temperature below 400 °C [34], leading to a fast conversion of Ag thin film to NPs. Consequently, this process could be more beneficial to form nanowires directly on the quartz substrate. However, as the thickness of Ag thin film increases (300 nm), the film requires a higher temperature (i.e., T > 800 °C) to shrink and form nanoparticles that enhance the growth of Ga_2_O_3_ as shown in Figure 8. The formation of nanoparticle is more sluggish with thicker Ag films, as more time and temperature are required to complete the dewetting of the film. At a constant temperature (1000 °C), the formation of a thicker nanocrystalline layer of Ga_2_O_3_ was increased at a shorter time and thinner and denser nanowires at a longer time.

The method of annealing thin films was used in the past to generate metal nanoparticles that act as a catalyst for thin film or nanowire growth. Using this method, first, a silver thin film is deposited onto a substrate. When the substrate was heated, the thin film dewets forming silver nanoparticles (Ag NPs). The benefit of first using a thin film [35] instead of directly applying nanoparticles is that it maximizes the size and density of the nanoparticles.

- Oxidation Temperature

**Figure 9:**
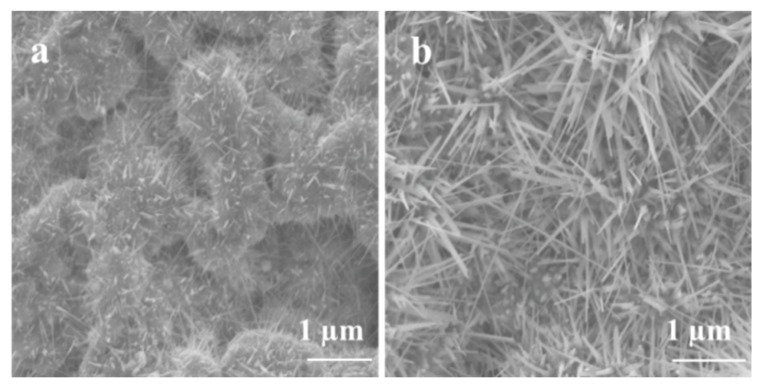
The effect of the oxidation temperature on the growth of Ga_2_O_3_ in the presence of 300 nm Ag catalyst. (**a**) 800 °C. (**b**) 1000 °C.

The Ga_2_O_3_ nucleation and growth of nanowires and nanocrystalline thin film on Ag catalyst could be influenced by temperature. In order to assess the effect of the Ag catalyst, Ag on GaAs was oxidized at different temperatures to measure the effect of Ag’s melting temperature (961.8 °C) on the growth of Ga_2_O_3_ nanowires and thin film. The results show that temperature is the major factor that serves to enhance the Ga_2_O_3_ growth mechanism due to the distribution of self-diffusion of Ag NPs, leading to increase the absorption of oxygen. The growth of Ga_2_O_3_ nanowires at 800 °C and 1000 °C with constant oxygen supply and sputtering 300 nm Ag thin film at the surface of liquid gallium on quartz substrate showed a remarkable growth of Ga_2_O_3_ nanowire for the first time via thermal oxidation (Figure 9). These results are in accordance with other reports [9,36] and it can be inferred that the impacts of the silver catalyst are related to the elevated temperatures. Figure 9 shows SEM images of Ga_2_O_3_ growth in the presence of 300 nm silver at 800 °C and 1000 °C for 60 min. High accumulation of nanocrystalline grains and short nanowires at 800 °C with diameters and lengths in the range of 500 nm–1 µm were observed at 800 °C; however, longer, denser, and thinner nanowires were obtained at 1000 °C compared to 800 °C. The diameters of Ga_2_O_3_ NWs are in the range of tens of nanometers and the length can reach several tens of micrometers, which mainly depend on the temperature and Ag catalyst.

First of all, high oxygen diffusion at a high temperature can be attained by using Ag catalyst which resulted in enhanced nanowire growth. The diffusion coefficient of oxygen in silver thin film shows that Ag is a better catalyst for enhancing the density and length of Ga_2_O_3_ nanowires than other nanoparticle catalysts, such as Au [37,38,39], Fe [40], or Pt [41], due to its higher value, which means that silver has a higher tendency to absorb oxygen. Secondly, the solubility of oxygen in molten silver thin film can be increased by increasing its temperature above its melting point of 961.8 °C.

High temperatures could reduce the surface energy of Ag islands and increase the diffusion of Ag NPs due to the driving force at the surface. Increasing the droplet surface will increase oxygen adsorption and diffusion. In addition, it increases the mobility, agglomeration, and distribution of Ag NPs [31,42]. As a result, temperature has a great influence on the kinetics of the growth mechanism due to the physical properties of oxygen in Ag, such as its melting point, diffusivity, and solubility.

- Oxygen Concentration

**Figure 10:**
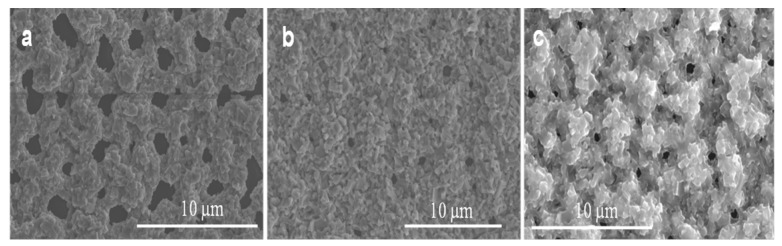
Growth of nanocrystalline grains of Ga_2_O_3_ under 300 nm Ag presence at different oxygen concentrations. (**a**) 0.088 mL/min. (**b**) 0.16 mL/min. (**c**) 0.28 mL/min.

**Figure 11:**
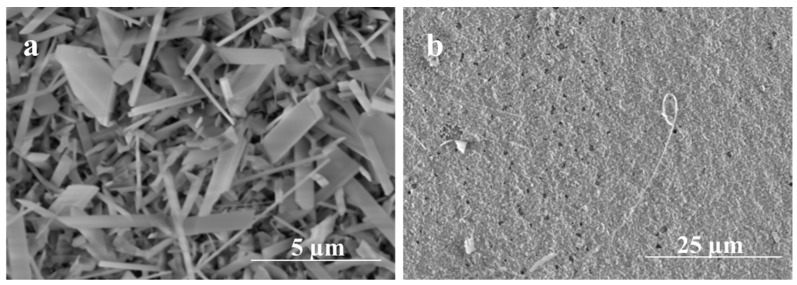
SEM images of Ga_2_O_3_ obtained at 1000 °C for 5 min. (**a**) Denser growth of nanowires using 5 nm Ag. (**b**) Nanocrystalline grains growth using 300 nm Ag.

The effect of oxygen flow rates on Ga_2_O_3_ growth at a constant annealing temperature (1000 °C) and time (10 min) has been investigated. Figure 10 shows SEM images of nanocrystalline Ga_2_O_3_ growth on quartz coated with 300 nm Ag thin film at 1000 °C and various oxygen concentrations and time. Obviously, the nucleation of nanocrystalline Ga_2_O_3_ was increased as the oxygen concentration increased form 0.088 mL/min to 0.28 mL/min at constant time, leading to thicker coating of nanocrystalline Ga_2_O_3_ on quartz substrate (Figure 10a,b). The growth of Ga_2_O_3_ was accomplished under a very low oxygen flow rate. Then the number density became much higher when the flow rate increased to 0.16 mL/min. However, after additionally increasing the flow rate to 0.28 mL/min, the nanocrystalline growth was increased and the film was still showing large crystallites. As the oxygen concentration and time increased from 0.088 mL/min to 0.28 mL/min, the accumulation of grown nanocrystalline Ga_2_O_3_ was slightly controlled, forming improved coating and growth of Ga_2_O_3_ on the surface of the quartz (Figure 10a,c). From these results, it could be expected that Ga_2_O_3_ requires more time to complete the growth from a nanocrystalline film in a short time (Figure 11) and that the nanowire nucleation will take longer (Figure 9b). Alternatively, these results may suggest that the nanowire growth observed at 300 nm silver films self-nucleates on the crystallites formed from a thick Ag film.

The grain size plays an important role in Ga_2_O_3_ nanowire growth. Specifically, Ga_2_O_3_ nanowires with much higher number density and aspect ratio (longer and thinner nanowires) were achieved from nanocrystalline Ga_2_O_3_. Besides, other factors such as surface morphology and shape of Ag catalyst may affect the nucleation and/or diffusion of Ga_2_O_3_ nanocrystalline and nanowire growth. The catalyst sets the nucleation spot and drives the one-dimensionality of Ga_2_O_3_ nanowire growth (Figure 3b and Figure 9). Due to the difficulty to control these factors, other parameters such as temperature, time, atmosphere, and Ga_2_O_3_ grain size are considered in the growth mechanism.

The influence of the oxidation atmosphere has significant impacts on the growth of nanowire due to the presence of Ag thin film. It has been observed that the motion of Ag NPs is influenced by the gas flow. The heated Ag films in the N_2_ gas dewet faster than the film in the Ar gas due to the molecular mass of N_2_ compared to Ar [34], leading to faster diffusion of Ag atoms. The higher mass of Ar limits diffusion through it, changing the growth dynamics. Furthermore, heating Ag NPs in an O_2_ gas as opposed to vacuum increases the surface self-diffusion of Ag atoms. Hence, the diffusion mechanism of Ag NPs enhances the growth of nanocrystalline thin film.

- Oxidation Time

To get a better understanding of the initial nucleation and growth of Ga_2_O_3_ on the surface of the quartz, very short heating times were performed to observe the morphology and composition of the short-growth material. As seen in Figure 11, for a growth time of 5 min, the influence of 5 nm Ag thin film catalyst produces a dense forest-like film of short nanowire; however, the film appears to have a more compacted mixture of Ga_2_O_3_ crystallites and silver at 300 nm Ag thin film. This result suggests that the nucleation and growth of Ga_2_O_3_ at 300 nm Ag is more sluggish, as would be expected from the more difficult dewetting behavior of 300 nm Ag films. On the other hand, the 5 nm Ag film rapidly dewets and nucleates, producing dense nanowires that grow early in the oxidation process.

- Growth Mechanism

**Figure 12:**
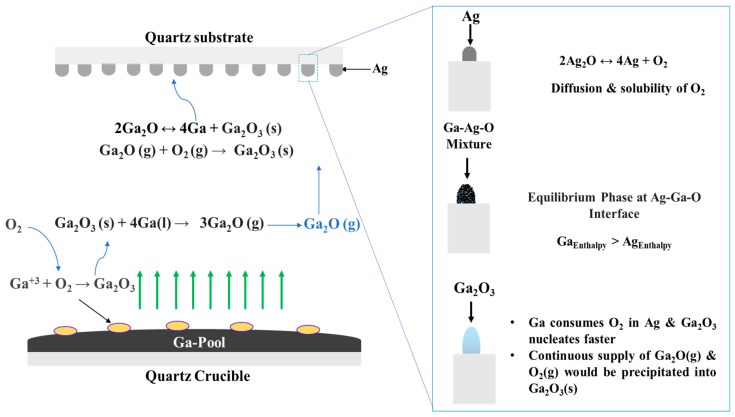
Schematic illustration of the proposed growth mechanism of Ga_2_O_3_ on the Ag-patterned quartz surface.

The use of a metal catalyst adds a new dynamic that contributes to enhancing the growth kinetics of Ga_2_O_3_ nanostructures due to its simultaneous interactions with O_2_, Ag, and the Ga_2_O_3_ surface. The growth mechanism is summarized in Figure 12. In general, there is a lack of experimental results that discuss the chemical and physical interactions between Ga and O. However, it is well known that the solubility of oxygen in liquid Ga increases with increasing oxidation temperature [43]. At higher temperatures, Ga forms gallium suboxide vapor (Ga_2_O) in the presence of oxygen (Figure 12). This vapor dissociates into liquid Ga and Ga_2_O_3_ on the Ag-patterned quartz surface. At temperatures above 200 °C, Ag_2_O dissociates into Ag and O_2_.

There are many reasons that could explain the oxygen interaction at the O and Ag interface. First, the silver catalyst increases the rate of oxygen adsorption with increasing temperature [44]. Originally, O_2_ atoms are adsorbed in Ag catalyst to form surface atomic oxygen and desorbed as O_2_ or diffused by volume diffusion. At 1000 °C, as O_2_ diffusivity and solubility into Ag is increased, liquid Ga strips oxygen from Ag at the liquid interface of the molten Ga and Ag mixture. The adsorption of O_2_ in the presence of dissolved Ga leads to the formation of the solid Ga_2_O_3_ surface. Eutectic reactions become possible with increasing temperature, and the interface changes from solid to a liquid–solid interface, which plays an important role in the supersaturation of the liquid–solid solution. The shape of the nanostructures is determined according to the nuclei surface formed on oxygenated gallium species [45] and Ag nanoparticles [46]. Different factors could determine the shape of the formed nanostructures such as pattern formation and time of coalescence, temperature, catalyst properties, etc. However, the degree of supersaturation is the dominant factor that controls the morphology of the growth.

- Electrical Properties of the Ga_2_O_3_ Film and Nanowires

**Figure 13:**
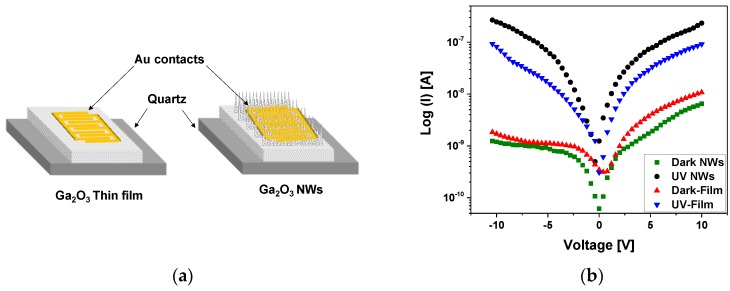
(**a**) Schematic of Au/β-Ga_2_O_3_/Au metal-semiconductor-metal (MSM) photoconductor of β-Ga_2_O_3_ thin film (left) and nanowires (right) on quartz. (**b**) Semi-logarithmic plot of current-voltage for Au/β-Ga_2_O_3_/Au MSM, including β-Ga_2_O_3_ thin film and nanowires, versus applied voltage characteristics at 10 V without and with UV illumination at 10 V.

The electrical contact consisted of 10 nm Cr and 150 nm Au and was sputtered on the gallium oxide surface to measure the electrical conductivity and the photocurrent response of β-Ga_2_O_3_ thin film and nanowires on quartz (Figure 13a). A custom probe station attached to a Keithly 2400 SMU was used with a UV light intensity of 15 W/cm^2^. The current-voltage (I–V) characteristics were measured at 10 V in dark conditions and under UV illumination (Figure 13b). Photocurrents were comparable to the dark current. The photoconductivity mechanism of the β-Ga_2_O_3_ NWs is credited to surface oxygen adsorption and desorption process [47], which is highly influenced by the presence of silver as a catalyst, leading to improved oxygen detection and hence the electrical properties of the β-Ga_2_O_3_ nanowires.

The photo-to-dark current ratio was much higher for the nanowires compared to the film. The photocurrent of nanowires was in the range of 2.33 × 10^−7^ A, which is one order of magnitude higher than that of thin film (9.16 × 10^−8^ A). The trap states of oxygen generated at the surface of Ga_2_O_3_ have a large impact on the photodetector performance [48]. Due to the large surface-to-volume ratio of nanowires and the existence of Ag NPs, the surface of NWs with trapped oxygen becomes highly sensitive. Nanowires as compared to thin films have a higher density of exposed surface states due to the dangling bonds at the surface.

## 4. Conclusions

A comprehensive study of the effect of silver thin film as an effective catalyst into the growth of Ga_2_O_3_ nanostructures was performed. To better understand the role of Ag in the growth dynamics and properties of the Ga_2_O_3_ film and nanowires, an Ag-patterned quartz surface was used, which offered the opportunity to investigate concomitantly the growth of gallium oxide on Ag-coated and uncoated surfaces. In this study, different parameters have been explored such as oxidation temperatures, oxygen concentration, oxidation time, and silver thin film thickness. Silver as a catalyst material for the growth of Ga_2_O_3_ has shown a great potential for high scale production. Oxygen solubility and diffusivity into silver is high and plays a critical role in enhancing the growth mechanism of Ga_2_O_3_. Our results could offer a simple, low-cost, and promising technique to grow thin film or nanowires for wide optoelectronics and sensing applications.
